# Changes to Sabah’s orangutan population in recent times: 2002–2017

**DOI:** 10.1371/journal.pone.0218819

**Published:** 2019-07-17

**Authors:** Donna Simon, Glyn Davies, Marc Ancrenaz

**Affiliations:** 1 WWF-Malaysia, Kota Kinabalu, Sabah, Malaysia; 2 Durrell Institute of Conservation and Ecology (DICE), University of Kent, Canterbury, United Kingdom; 3 HUTAN-Kinabatangan Orangutan Conservation Programme, Sandakan, Sabah, Malaysia; 4 Borneo Futures, Bandar Seri Begawan, Brunei Darussalam; San Diego Zoo Institute for Conservation Research, UNITED STATES

## Abstract

The Bornean orangutan is critically endangered and monitoring its population is needed to inform effective conservation management. In this paper, we present results of 2014–17 aerial nest surveys of the major orangutan populations in Sabah and compare them with baseline data produced during surveys conducted in 2002–03 using similar methods. Our results show three important points: a) by increasing the survey effort (estimated at 15–25% cover), sparsely scattered orangutan sub-populations not recorded in the previous aerial surveys were located and the accuracy of the nest count estimates is expected to improve; b) large populations in the interior forests of Sabah, occupying sustainably managed logged and unlogged forests, have been stable over 15 years and are of vital importance for the species’ conservation; c) fragmented populations located in eastern Sabah, that are surrounded by extensive oil palm plantations, have declined at varying rates.

## Introduction

The Bornean orangutan (*Pongo pygmeaus*) is critically endangered [1], with recent studies indicating that population declines may be more rapid than previously thought [2,3]. A recent modeling study estimated that more than 100,000 Bornean orangutans have died in the past 16 years [4], with habitat loss, fragmentation and hunting being the prime drivers for population declines [5–8]. Across Borneo, between 1973 and 2010, 39% of rainforests were lost, including 98,730 km^2^ of prime orangutan habitat [9]. Under a “business-as-usual” trend, it is estimated that a further 37% of suitable orangutan habitat (155,106 km^2^) will be converted to oil palm and tree plantations between 2010 and 2025, which would account for an additional loss of 57,140 km^2^ of orangutan habitat [8]. Impacts of climate change are also likely to significantly reduce habitat, leading to gradual population declines [10–12]. Hunting pressure, either for meat or in conflict situations with humans [13] further accelerates this population decline by causing the deaths of several thousand orangutans annually, half of which end up being eaten [7].

In Sabah (73,620km^2^), located in the northern part of Malaysian Borneo, the distribution and population densities of orangutans (*P*.*pygmaeus morio*) have been surveyed extensively and documented for many years [14–18]. Through intensive ground surveys for individuals and nests, WWF-Malaysia estimated 4,000 orangutans in 5,000 km^2^ of Sabah’s primary forest in the eastern lowlands and central uplands in the early-1980s [15,16]. Subsequently, in the mid-1980s, the first aerial nest surveys from helicopter were conducted and combined with habitat assessments (survey area: 28, 872 km^2^) which gave an estimate of up to 21,000 orangutans in forest reserves and state parks [17]. Then, 15 years later, another state-wide survey was conducted in 2002–03, using a combination of aerial and ground nest surveys (survey area: 18 470 km^2^), estimating 11,000 orangutans in 16 major orangutan populations [18]. In all these studies, no survey was conducted outside of protected areas (forest reserves, wildlife reserves and sanctuary and state parks), in mangrove areas, or in agricultural landscapes. Therefore, the total number of orangutans in the whole State is expected to have been higher than reported.

Three main factors need to be considered in the context for the current study: hunting, forest degradation through timber extraction and deforestation for agriculture. In most parts of western and northern Sabah, such as Crocker Range, Upper Sugut or Pitas area, there has been evidence of hunting by traditional communities, which reduced orangutan populations in the 1970s [15,17] up till the early 2000s. The scarcity of orangutan populations in the south-west of Sabah is also a result of hunting in a combination with the effects of higher altitude [17]. Meanwhile, in the central and eastern forest of Sabah, little hunting of orangutans has been recorded since the 1970s [15,17]. At present, although hunting and killing of orangutan is not common in Sabah, the Sabah Government revised and imposed a heavier penalty for offenders under the Sabah Wildlife Enactment 1997, in an effort to deter hunting and killing of totally protected species including orangutans.

Lowland rainforests in Sabah are the most important habitat for orangutans. However, this is also where logging was more intensive in the 70s-80s, resulting in an extreme degree of damage to forest structure, affecting its capacity to regenerate naturally and exhausting Sabah’s timber stock [19]. In order to manage and control timber production in Sabah, sustainable forestry management (SFM) was gradually introduced and implemented in Sabah’s forest reserves since 1997 [20]. The SFM practices are more compatible with the long-term survival of wild orangutan [21], with evidence suggesting that their numbers can return to pre-logging levels in the eastern lowlands and central uplands, once the logging stops [22,23]. This particularly applies where reduced impact logging (RIL) and Forestry Stewardship Council (FSC) certified timber production are practiced. Since its implementation, 1.56 million ha (93%) of Sabah State’s commercial forest reserves practiced SFM and almost half (48%) of these SFM forests are already under various forms of certifications [20].

The complete loss of forests to agriculture and fire, however, does lead a decline in orangutan populations. In the early 1980s, State government policy was to convert large areas of forest on the alluvial lowland soils in eastern Sabah to croplands [17,19], originally for cacao plantation. In the mid-1990s, oil palm became the preferred crop, replacing cacao, which now covers an area of 15,500 km^2^ [19]. It is estimated that more than 10,000 orangutans were lost during the period of 1980–2000 when land clearance activity was at its peak [17,19].

Given these losses in recent times, it is important to assess whether Sabah’s orangutan populations have remained stable in large areas of logged forest and how populations are surviving within large agricultural landscapes. The objectives of the current study were therefore to: 1) assess orangutan population trends in Sabah over a 15-year period by comparing current estimates with a baseline established in 2002–03 [18] and 2) assess how the recently enlarged network of Totally Protected Areas (TPAs) which includes logged and unlogged forests, contributes to orangutan conservation.

## Material and methods

### Study area

**Fig 1:**
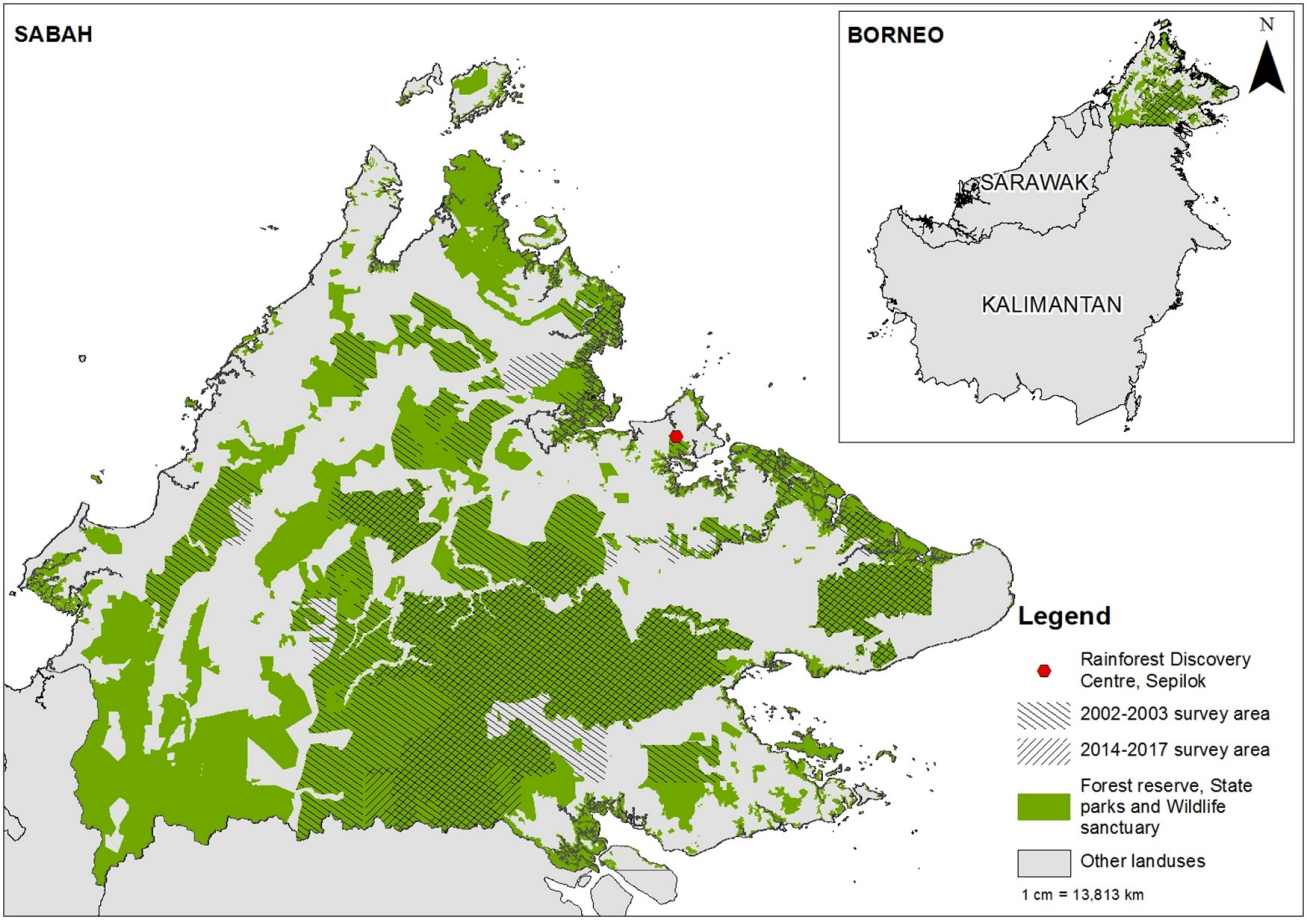
Comparisons of areas covered during the 2002–03 survey and the 2014–17 surveys that are the subject of this paper.

Just under half of Sabah’s landmass has been gazetted as permanent forest reserve (PFR) (3.54 million ha), where 1.61 million ha of these forests are protected and the remaining 1.93 million ha are production forest [20]. In addition to PFR, State parks, wildlife sanctuaries and wildlife conservation areas added another 0.27 million ha of protected forests [20]. Within these forests, the 2002–03 orangutan population surveys had identified 16 major orangutan populations [18] and 15 years later WWF-Malaysia conducted comparable aerial nest surveys in eight major orangutan populations, equivalent to 63% of the previously surveyed areas (1.16 million ha), over a 4-year period: 2014–17 (Fig 1) and the results are reported here. We excluded areas within forest reserves that had been converted to oil palm plantation and industrial tree plantation, such as *Acacia* and rubber. For safety reasons with the helicopter, we also avoided steep slopes.

### Survey methodology

Assessing the population status of animals with large home ranges and living at low densities, especially in rainforests, are often difficult. So, great ape is typically surveyed from the ground or from the air, by counting the nests they build for sleeping and resting [18,24,25]. We conducted aerial orangutan nest count using helicopters, with the survey methodology and data analysis closely following the earlier set of surveys conducted in 2002–03 [18], to allow comparison between the two studies. However, some differences do apply, as shown in Table 1. Most importantly, the current study has used a greater sampling effort, calculated at 15–25% cover. This study defined sampling effort as the ratio between observer’s maximum distance to detect nest (between 150-250m) and 2km spacing distance between parallel aerial transect. Furthermore, this survey gathered data from only eight (11,673km^2^) out of the 16 (18,470 km^2^) major orangutan populations surveyed in the 2002–03 survey [18].

**Table 1:** List of differences in aerial methodology between 2002–03 survey [1,8] and current 2014–17 survey.

Survey features	2002–03 survey		2014–17 survey
Number of survey regions	16	8^a^	8^a^
Survey period	Between 2002–03	Between 2002–03	Between 2014–17
Aerial transect length (km)	1963	Not available	5491
Orangutan habitat (km^2^)	18 470	13 280^b^	11 673
Sampling effort^c^	1.8–16.9%	2.3–3.6% and 8.0%	15–25%
Spacing of aerial transect	>2km	>2km	2 km
Data collection	recorded every 30 sec	Recorded every 30 seconds	continuously tagged with Garmin GPSMAP 62s
Ground survey	Yes	Yes	None
Habitat calibration^d^	0.54 in exploited swamp forest, 1 in logged forest and 1.5 for primary forest	0.54 in exploited swamp forest, 1 in logged forest and 1.5 for primary forest	Same habitat calibration used in 2002–03 survey
Assessment of forest type	Recorded during survey	Recorded during survey	From satellite imagery analysis

^a^Same eight regions surveyed in both study periods.

^b^Slightly larger than current study because forest conversion in part of the area took place after 2002–03 survey.

^c^Ratio between observer’s maximum distance to detect nest (between 150-250m) and 2km spacing distance between parallel aerial transect.

^d^Habitat calibration is generated based on relation of nest density estimated from the ground with aerial index (number of nest per km flight) from aerial survey [18].

We used a systematic stratified sampling technique, with parallel line transects averaging 2km spaced apart and by randomly selecting the first line. The aerial transect planning was conducted using Google Earth software; avoiding steep terrain. A small type Bell 206 Jet ranger helicopter 5-seater was used to carry out the surveys. A total of four persons conducted each survey: a pilot, a co-pilot and two nest observers (seated at the back and on either side of the aircraft). All observers had at least 5 years’ experience of surveying nest from the ground, and only the left observer (L) had 10 years’ experience in aerial nest counts. The pilot ensured helicopter speed was kept at about 70 km/h and maintained 60 to 80m height above the forest canopy for best nest observation. The co-pilot recorded the actual transect using a handheld global positioning system (GPS) and ensured the pilot followed the transect plan. In all, 5491km of transects were flown, with an average length of 6.28km, for the 874 transects flown. Sometimes, the pilot had to navigate away from the planned transect to avoid emergent trees, strong winds and sudden steep climbs. The co-pilot also informed the nest observers when to start or stop nest counting.

Each nest observer looked for nests on one side of the helicopter and continuously recorded each sighting onto their GPS as the survey progressed, regardless of its decay status (e.g. recently made or old) and as long as nests were still visible. Travelling at fast speed and counting nests under difficult observation conditions as they passed, meant that it was not feasible to measure the sighting distances from the observer to the nest to calculate strip-width and to note on each nest’s decay status. Garmin GPSMAP 62s type GPS was used to record each sightings, as it allows observer to easily press on the tag waypoint button without looking at GPS.

To assess the sampling effort, a field test was conducted at Rainforest Discovery Centre Sepilok (Fig 1), where the same observer’s ability to detect nests from canopy walkways in the tree crown was measured using a Nikon Forestry Pro Rangefinder. This indicated that nests were detected up to 250m away and if this is extended to survey strip-widths when flying, the survey effort would equate to approximately 25% of the surveyed forest reserve searched for orangutan nests. Given the many challenges of detecting nests from a moving helicopter, a more conservative effective strip-width might be 150m [18] which gives 15% sampling effort. In the end, strip widths were not used directly to calculate population densities as nest density was calculated through the calibration between ground surveys and aerial surveys [18].

### Statistical analyses

#### Observer’s correction factor

We assessed the differences in observer’s nest detection skills, as this has been noted by previous studies in Sabah [17,18] and applied a correction factor whenever there was a substantial disparity (Table 2). In this survey, as previously mentioned in the survey methodology section, only L had prior experience with aerial nest surveys. Therefore, it is unsurprising that L spotted more nests than R_1_ (1^st^ right observer) and R_2_ (2^nd^ right observer). Aerial nest observation skills were known to improve over time, so the correction factors for R_1_ and R_2_ were applied only on areas that showed there is a significant difference between L and R. Wilcoxon Signed rank was used for significance testing [26]. Formula for correction factor is [(1 − R: L nest ratio)x 100%]. Assuming a random nest distribution in the forest, we estimated that the nests missed out by R_1_ and R_2_ represented 11% and 41% of the total number of nests recorded by L. The corresponding correction factors were applied on R_1_ and R_2_, as shown in Table 2.

**Table 2:** Number of nests detected by *L* and *R* after applying observer’s correction factor (the remaining 19 forest reserves were not corrected).

No.	Forest reserve			Number of nests			Total nest
		L	R_1_	N	R_2_	N	
1	^a^Ulu Segama	9650	10308	8363	-		19958
2	^a^Malua	5234	4653	3775	-		9887
3	^a^Northern Kuamut	4145	3886	3153	-		8031
4	^a^Kuamut	1683	1625	1468	-		3308
5	^a^Trusan Sugut	927	632	513	-		1559
6	^a^Kalabakan	547	279	262	-		826
7	^a^Trus Madi (half east)	268	153	124	-		421
8	^a^Maliau Buffer	246	205	166	-		451
9	^a^Silabukan	128	51	41	-		179
10	^a^Malubuk	42	30	24	-		72
11	^a^Sapulut	38	3	3	-		42
12	^a^Burod-Urod	2	2	2	-		4
14	^b^ Kulamba	1137	-		874	620	2011
15	^b^ Tabin	5799	-		4943	3508	10742
16	^b^ Mt Hatton	295	-		300	213	595
17	^b^ Trus Madi (half west)	175	-		42	30	217
18	^b^ Kuala Meruap	128	-		80	57	208
19	^b^ Bukit Taviu	71	-		96	68	167
20	^b^ Nuluhon Trus Madi	5	-		3	2	8

^a^correction factor of 11%

^b^correction factor of 41%

#### Estimating nest density from aerial index (AI)

An aerial index (AI) was calculated using the following formula, AI = (nL + nR)/2 where AI is the number of nests per km, nL and nR are the number of nests detected by L and R. The AI was calculated per study area. Habitat calibrations were then applied on selected AI, to calibrate nest detectability; as it is known to fluctuate according to habitat type [18]. For example, a nest is more visible in degraded forest and in swamp forest compared to primary forest because of the canopy opening. For our analysis, we are using the same habitat calibrations that had been designed in Sabah by comparing ground and aerial survey results [18]. A 0.54 habitat calibration function was applied to AI in heavily degraded forest, such as in Bukit Piton and Northern Kuamut (west), to correct the increased aerial nest detectability due to canopy openness, comparable with the heavily degraded forests in the Lower Kinabatangan [18,21]. A 1.5 habitat calibration function was applied to AI for Danum Valley, because nests in primary forest tend to be less visible due to the closed canopy [18]. Then, to estimate nest density (Dˢ), the formula D^s^ = exp [4.7297 + 0.9796 Log (AI)] was used [18].

#### Calculating orangutan density (Ď) and confidence interval (CI)

The final step in the population density calculation is to determine the relationship between number of orangutan nests in a forest, and corresponding number of individual orangutans. The orangutan density (Ď) estimate was obtained by converting D^s^ using the formula Ď = (D^s^/P x R x T) where, P is the proportion of nest builders in the population, estimated as 0.9 (as young infants do not make nests) for Bornean orangutans, R is 1.084 for the daily rate of nest production, T value used is 286.3 days [18]. The R value and T value used in this study has been studied for two Bornean orangutan population in Kinabatangan (R = 1,005; T = 258 days) and Gunung Palung (R = 1.163; T = 399 days). Thus, the average between these two values from two locations were used for establishing the baseline data in 2002–03[18]. We are using the same value in order to compare any population change between the two surveys. Then the CI for the Ď was calculated by using the formula D^s^/C for lower CI and D^s^.C for upper CI Where $C=exp(0.6067\;x\sqrt{1+v*})$ and v* = 0.1908 − 0.2628 x Log (AI) + 0.1132[log(AI)]2 [18]. This model was created by correlating the results of a series of ground estimates obtained in the various forests of the Lower Kinabatangan and other areas with the results of helicopter surveys obtained above the same forests [18].

## Results and discussion

**Fig 2:**
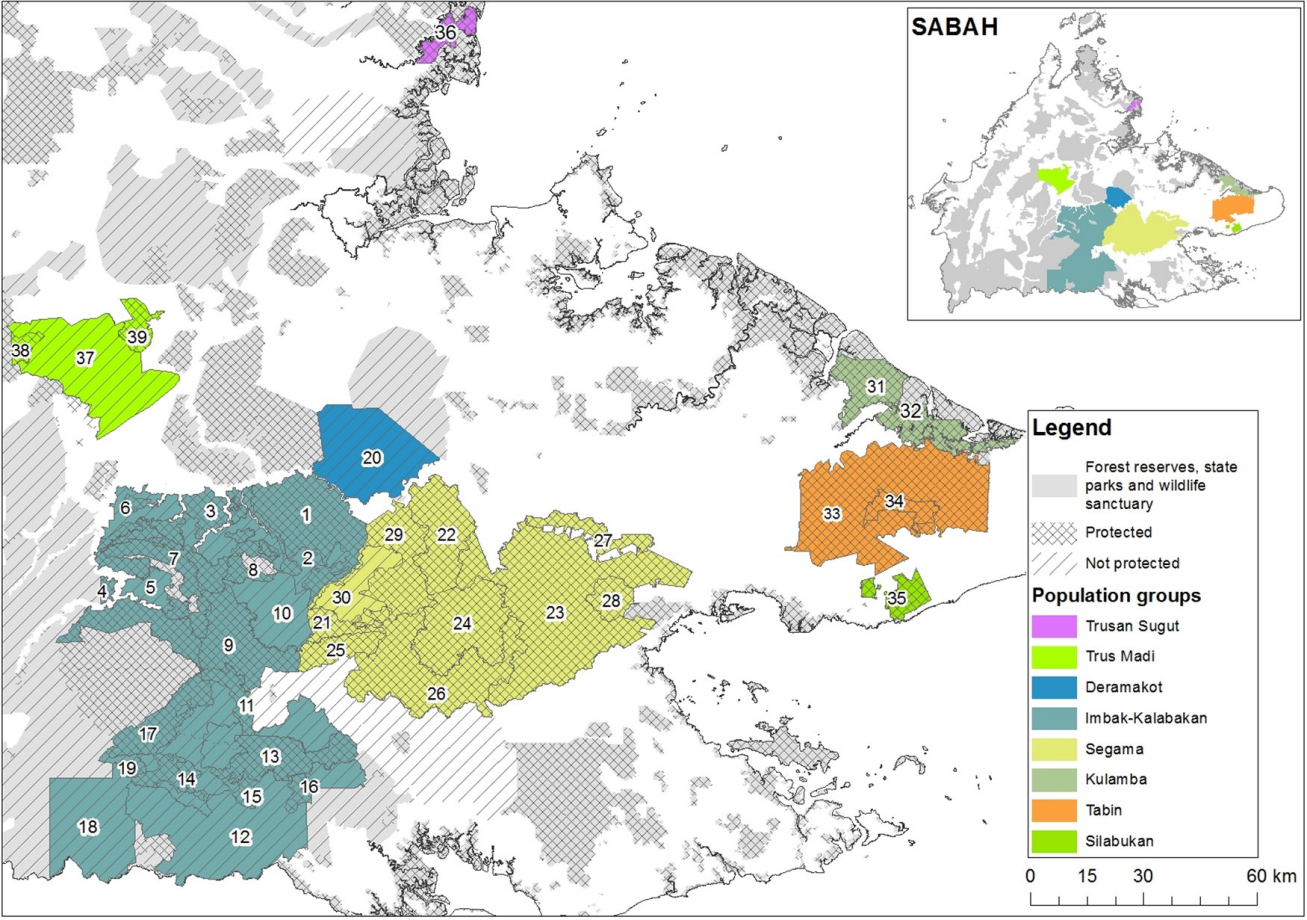
Orangutan populations based in forest reserves surveyed 2014–17.

We carried out nest surveys along 874 aerial transects totaling 5,491 km, between May 2014 and March 2017 representing an overall sampling effort covering 15–25% of the survey area. A total of 96,580 nests were recorded, which equates to an overall population estimate of 9,558 orangutans (95% confidence interval: 6,815 to 15,129). Table 3 and Fig 2 presents the orangutan density estimates and populations sizes for the eight major orangutan populations and the forest reserves within them. Considering the distribution of orangutan nests, along each transect (S1–S8 Figs) and the current population estimates, three important themes emerged.

**Table 3:** Orangutan population density estimates according to forest reserves (S1–S8 Figs).

No	Forest Reserve	Distance of Aerial transect (km)	Total nest	AI (nest/km)	Orangutan per km^2^ (95% CI)	Size of Habitat (km^2^)	Orangutan Population size (95% CI)
**Imbak-Kalabakan**							
1	**Northern Kuamut (West)	64.03	207	3.16	0.38 (0.25–0.58)	504.69	193(128–292)
2	**Kuamut (West)	28.09	24	0.42	0.28(0.18–0.43)	72.86	20(13–31)
3	Sg Imbak Bufferzone	160.46	189	0.59	0.32(0.21–0.49)	192.26	62(41–94)
4	Sg Lulunguyon	5.85	15	1.28	0.45(0.30–0.68)	20.58	9(6–14)
5	Sg Pinangah	187.82	84	0.22	0.21(0.14–0.33)	390.87	84(55–129)
6	Sg Ayop	7.16	0	0	0	15.5	0
7	Imbak Canyon (U)	55.93	16	0.14	0.18(0.11–0.27)	133.61	24(15–37)
8	Sg Imbak	43.57	49	0.56	0.32(0.21–0.48)	126.18	40(26–61)
9	Mt Magdalena (West)	159.15	642	2.02	0.55(0.37–0.82)	350.91	192(128–287)
10	Gunung Rara	272.71	522	0.96	0.40(0.27–0.60)	601.48	240(160–362)
11	Gunung Rara Corridor	58.68	13	0.11	0.16(0.10–0.25)	89.22	14(9–22)
12	Kalabakan	345.03	798	1.16	0.43(0.29–0.65)	981.99	424(282–637)
13	Sg Tiagau and Ext	145.66	220	0.76	0.36(0.24–0.54)	269.16	97(64–146)
14	Tambulanan	12.92	2	0.08	0.14(0.09–0.21)	32.77	4(3–7)
15	Sg Anjeran Jemut	15.66	2	0.06	0.13(0.08–0.20)	37.5	5(3–7)
16	Sg Sumagas	17.74	9	0.24	0.22(0.15–0.34)	41.96	9(6–14)
17	Maliau Buffer	223.65	430	0.96	0.40(0.27–0.60)	344.62	138(91–207)
18	Sapulut	134.06	41	0.15	0.18(0.12–0.28)	583.07	107(69–165)
19	Nurod-Urod	8.25	4	0.26	0.23(0.15–0.35)	16.51	4(2–6)
**Deramakot**							
20	Deramakot	270.18	13790	25.52	1.61(1.08–2.39)	550.83	887(597–1318)
**Segama**							
21	Northern Gunung Rara	21.64	753	17.4	1.37(0.92–2.03)	58.85	81(54–120)
22	Malua	150.09	9424	31.4	1.76(1.18–2.61)	339.54	597(402–888)
23	Ulu Segama	631.57	18933	14.99	1.28(0.87–1.91)	1272.35	1634(1101–2426)
24	*Danum Valley (U)	218.87	5085	11.62	1.37(0.92–2.03)	438.68	601(405–892)
25	Mt Magdalena (East)	68.79	2063	14.99	1.28(0.87–1.91)	132	170(114–252)
26	Mt Louisa	311.32	10874	17.46	1.37(0.92–2.03)	642.36	880(593–1307)
27	**Bukit Piton	114.7	8311	36.23	1.44(0.97–2.14)	121.64	176(119–261)
28	Sg Taliwas	50.43	1281	12.7	1.20(0.81–1.78)	97.13	116(78–173)
29	Northern Kuamut (East)	171	7506	9.99	1.08(0.73–1.60)	385.55	417(281–619)
30	Kuamut (East)	95.98	3122	16.27	1.33(0.90–1.97)	203.56	271(182–402)
**Kulamba**							
31	Kulamba	77.73	1995	12.83	1.20(0.81–1.78)	203.83	245(165–364)
32	Kuala Meruap	81.65	208	1.28	0.45(0.30–0.68)	183.71	83(55–124)
**Tabin**							
33	Tabin	558.65	10745	9.62	1.06(0.72–1.58)	1123.96	1195(805–1775)
34	Mt Hatton (U)	43.59	595	6.83	0.92(0.62–1.37)	89.68	82(55–123)
**Silabukan**							
35	Silabukan	57.79	174	1.5	0.48(0.32–0.72)	104.95	51(34–76)
**Trusan Sugut**							
36	Trusan Sugut	42.01	1496	17.81	1.38(0.93–2.05)	85.34	118 (79–175)
**Trus Madi**							
37	Trus Madi	317.9	623	0.98	0.40(0.27–0.61)	676.09	272(181–410)
38	Nuluhon Trus Madi	21.68	8	0.18	0.20(0.13–0.30)	41.93	8 (5–13)
39	Bukit Taviu	39.1	106	1.35	0.46(0.31–0.69)	86.17	40 (26–60)

Unlogged forest denoted as (U)

*0.54 habitat correction were applied for primary forest

** 1.5 habitat correction were applied for over degraded forest

First, in the Imbak-Kalabakan region (5,460 km^2^), the study has uncovered a substantial population of about 1,770 orangutans. This region has a mixture of heath, lowland and upland mixed dipterocarp forest types; in which there is a sparse, scattered and widely dispersed set of orangutan nests that suggests widely separated, small sub-populations (S1–S8 Figs). There is possibly a small concentration of orangutans in the eastern boundary next to an area of forest that was cleared for agriculture. Overall, this region contributes to more than 10% of the total orangutan population estimated in the current study.

Second, the Deramakot and Segama regions (4241 km^2^), which are adjacent to each other, sustained the largest orangutan populations in Sabah, amounting to about 6,000 orangutans. This region is therefore of greatest importance for the conservation of Sabah’s wild orangutans.

Third, three populations surveyed in the eastern lowlands of Sabah (1705 km^2^) account for about 1,600 orangutans, and are all ‘pressed-in’ by surrounding large-scale and long-established oil palm plantations (i.e. Kulamba, Tabin and Silabukan). A small population ofabout 110 orangutans are found in Trusan Sugut (85 km^2^) on the north-east coast, and the remaining about 300 orangutans are found in the hilly, sub-montane forests of Trus Madi (804 km^2^).

### Sampling intensity

The large sampling intensity of this study, (15–25% survey effort) is the highest survey effort ever reported for great ape species [24], thus allowing two important improvements. First, it allows a helpful visual interpretation of population distribution, which can in turn inform our understanding of population movements, as well as identify areas for conservation intervention (S1–S8 Figs). Second, large tracks of forest in the Imbak-Kalabakan region, which are remote and have low orangutan population densities allow a much better population estimate showing the location of a substantial but scattered population of orangutans that had been underestimated in the past.

This level of survey sampling is inevitably costly. The helicopter hire cost was about Malaysian Ringgit (RM) 70 per minute (US$18 per minute). Overall, the whole orangutan survey activities spread over four years amounted to a total cost of RM 485,000 (US$ 120K). Although aerial surveys proved to be costly, extensive areas could be surveyed within a relatively short time. Additionally, aerial survey is an efficient tool for population assessments, as well as for monitoring encroachment, illegal logging, illegal mining, etc. It is important that these surveys are repeated every 5 to 10 years to monitor population trends, and hopefully less expensive techniques, such as drone technology or remote sensing, will improve and can be used in the future [27].

### Comparing orangutan populations over 15 years (2002–03 to 2014–17 survey)

We compared the current results with the same regions surveyed in the 2002–03 survey (Table 4), noting both population density, as well as the absolute population figures, when interpreting the patterns of change. The current survey area was 5% less than the previous study, almost entirely because of deforestation in two areas: Trusan Sugut (then labeled as Bonggaya) and Imbak-Kalabakan (then labeled as Kuamut–inclusive of Benta-Wawasan area).

**Table 4:** Comparisons of orangutan population density estimates for eight major orangutan populations in 2002–03 surveys and this study.

			Size of habitat (km^2^)		Orangutan per km^2^ (95% CI)		Orangutan population size (95% CI)	
No.	Region	Status	2002–03	2014–17	2002–03	2014–17	2002–03	2014–17
1	*Imbak-Kalabakan	PF	860	2175	0.06 (0.02–0.19)	0.32 (0.21–0.48)	51(17–166)	694 (458–1051)
		P	4600	2630	0.06 (0.02–0.19)	0.33 (0.22–0.50)	262(80–860)	876 (579–1323)
2	Deramakot	P	530	550	1.50 (0.55–4.05)	1.61 (1.08–2.38)	792(292–2148)	887 (597–1317)
3	Segama	PF	480	3488	1.04 (0.38–2.81)	1.37 (0.92–2.03)	498 (183–1350)	4775 (3216–7089)
		P	3150	204	1.30 (0.49–3.51)	1.33 (0.9–1.97)	4086 (1508–11073)	271(182–402)
4	Kulamba	PF	>170	387	2.50 (0.91–6.85)	0.93(0.67–1.48)	500 (182–1369)	361(223–488)
5	Tabin	PF	1110	1213	1.26 (0.47–3.42)	0.99 (0.67–1.48)	1401 (517–3796)	1207(813–1794)
6	Silabukan	PF	100	105	0.58 (0.21–0.59)	0.48 (0.32–0.72)	58 (21–159)	51 (34–76)
7	**Trusan Sugut	PF	600	85	0.18 (0.06–0.54)	1.38 (0.93–2.05)	111(38–324)	118 (79–175)
8	Trus Madi	PF	80	128	0.46 (0.17–1.28)	0.36 (0.24–0.55)	37(13–102)	46 (31–70)
		P	600	676	0.41 (0.15–1.14)	0.40(0.27–0.61)	245(88–682)	272 (181–410)

*part of the Kuamut region in 2002–03 survey [18]

**part of the Bonggaya region in 2002–03 survey [18]

PF is Protected forest

P is Production forest

The first striking comparison is that the Imbak-Kalabakan region showed a tremendous increase in population size, from 313 to 1,570 orangutans. The population has remained sparse and dispersed over a large area. Some patches on the eastern boundary, with modestly higher population densities, may reflect immigration from adjacent land areas where there has been substantial clearance of forest between the survey periods. This accounts for only a small portion of the overall population and the larger population located recently is due to the intensive survey efforts in our study.

Second, the combined population of orangutans in Deramakot (part of the Upper Kinabatangan region in 2002–03) and Segama regions has remained stable over the past 15 years, from 5,376 to 5,933, where both have been managed under the SFM practices. This reinforces the conclusions of previous surveys carried out in the Segama region, which showed that orangutan populations can be maintained in well-managed logged forests [21], and emphasized the importance of the continuation of conservation management in the central forests of Sabah, for orangutans and other forest species. The other forest reserves within the Upper Kinabatangan region (e.g. Tawai, Tangkulap, Segaliud-Lokan) need to be re-surveyed to gauge the health of the entire orangutan population for this upland area, and thereby inform future forest management.

Third, less comfortable news comes from the eastern lowlands, where the fragmented populations in Kulamba (361) and Tabin (1,207) have declined by 30% and 15% respectively in 15 years, while the small population in adjacent Silabukan (51) has remarkably remained stable. An even higher rate of decline has been recorded by separate studies in the Lower Kinabatangan region, where 1,125 orangutans [18] declined to 800 orangutans [28] over 15 years. Taking these estimates together, a minimum of 650 orangutans were lost in the protected areas of eastern Sabah lowlands since 2002. These declines sound a conservation alert and emphasizes the need for population monitoring to assess whether this reflects a period of ‘settling down’ after populations were compressed into these areas by large scale plantation agriculture prior to the 2002–03 survey, or if this a longer term declining population trend within the protected forest themselves.

Fourth, the population estimate in Trusan Sugut had not changed much, (111 to 118 orangutans), but the population density had increased dramatically from 0.18 to 1.38 orangutans per km^2^. This near ten-fold increase for the area is probably a response to 400 km^2^ of forests being gradually converted to oil palm in the adjoining Bonggaya Forest Reserve. Many of the orangutans previously found here could have moved into the remaining forested areas, thus increasing the population density within the Trusan Sugut. This is analogous to the orangutan population movements away from active timber felling activities elsewhere in Sabah [21], and mirrors reports of Sabah orangutans becoming concentrated in small primary forest patches which remain after clearance for logging and agriculture in the 1980s [16]. This has been described as ‘refugee crowding’ in peat-swamp forest in Kalimantan [29].

Finally, the modest population in Trus Madi (46), has remained stable in these hilly, sub-montane forests, despite moderate disturbance from logging and conversion of some areas to rubber. Further surveys are needed to confirm population sizes and changes over time of the small or modest populations in other hilly or mountainous regions i.e. Crocker Range Park, Mount Kinabalu Park and Tawau Hills Park. It is also important to recall that orangutan numbers in forests that are outside of government gazette lands (i.e. PFR, State parks, sanctuaries and conservation area) have not been assessed in this or previous studies.

## Orangutan conservation in Sabah

The current population estimate for the eight regions surveyed between 2014–17 is 9,558 orangutans (95% confidence interval: 6,815 to 15,129), with separate studies recording a further 800 orangutans in the Lower Kinabatangan region [28]. This gives a conservative population estimate of 10,300 orangutans in Sabah’s PFR and wildlife sanctuary (at the present time. Figures from other populations measured in the 2002–03 survey [18], or even earlier [17], give an additional of 1,159 orangutans, but there are no recent surveys to indicate what changes have occurred. If the general trends from the re-surveyed areas reported here are extrapolated, orangutan populations in the central uplands are expected to remain stable as long as RIL and other protection measures are kept in place. In the eastern regions of Sabah, where most intensive agricultural activity has been concentrated over three decades, orangutan populations are expected to continue to decline, but at unknown rates.

Precise information is needed to inform the Sabah Government about conservation strategies. A network of Sabah’s Totally Protected Areas (TPAs) comprising: Class I-Protection Forest Reserves, Class VI-Virgin Jungle Reserve, Class VII-Wildlife Reserves, State Parks, Wildlife Sanctuaries and Wildlife Conservation area, now covers 1.9 million ha (Fig 2) and harbors more than 70% of the total orangutan population in Sabah. The area under TPAs have increased from 12% to 26% of Sabah’s landmass in 15 years, with many news ones being strongholds for orangutan. The bulk of Sabah’s orangutans are found in the central upland region (e.g. Segama, Deramakot, Imbak-Kalabakan), where there are estimated to be over 7,500 orangutans and where considerable strides have been made in supporting conservation. The majority of the TPAs comprise logged-over forest (S1 Table), without orangutan hunting, where orangutan populations remain healthy in a sustainably managed forest, following RIL and FSC standards [21]. State Parks (i.e. Kinabalu, Crocker Range, Tawau Hills) support far fewer orangutans [17].

In the eastern lowlands, the main orangutan conservation areas comprise of Tabin Wildlife Reserve (1,207) and Kinabatangan Wildlife Sanctuary (800) [21], followed by smaller populations in Kulamba (361), Trusan Sugut (118) and Silabukan (51). Tabin is the largest single block (1,213 km^2^) of forest, with the largest orangutan population, which requires special conservation attention to minimize human disturbance, and careful monitoring of the impact of any future orangutan translocations. Kinabatangan still harbors a large orangutan population, and is a very important area for eco-tourism. This sanctuary, as with other forest patches in the oil palm dominated landscapes, requires careful management of meta-populations through habitat management, and the establishment of ecological corridors that can facilitate gene flow for healthy breeding; allowing movement of individuals for social interactions, as well as keep options open to adapt to future climate change impacts [30].

These results from Sabah are important in informing current and future population estimates for Bornean orangutans [3,4]. The Sabah circumstances are distinctly different from many areas of Kalimantan [4], and so these recent figures allow refinement of models to estimate Bornean orangutan populations. They also provide more positive news for orangutans in this region of north-east Borneo. First, this comparative study of Sabah’s orangutans indicates that major population declines occurred during the 1980s-1990s, when deforestation rates for agriculture were very high. The exhaustions of Sabah’s timber and limited land for oil palm conversion in more recent times have slowed down the anthropogenic orangutan decline. Second, populations have maintained their numbers for at least 15 years, in areas of the eastern lowlands and central uplands where there are no apparent recent impacts of hunting. Third, the large-scale monoculture agricultural development in Sabah is distinct in that smallholder farmer only account for 15% of the oil palm planted area. Fourth, among the State government policy in Sabah are to ensure all timber production to be FSC-certified and oil palm production to be RSPO-certified, a bold move towards sustainability. Fifth, Sabah policy is to keep 50% of Sabah’s landmass forested with 30% being TPA.

Sabah remains the stronghold of Malaysia’s orangutan and supports internationally significant populations, which need on-going protection in forest areas. Monitoring of these populations, and those in agricultural landscapes where population fragmentation is a threat, is essential to inform further conservation action for this critically endangered species.

## Supporting information

S1 Fig Distribution of orangutan nests along 10km grid aerial transect in Imbak-Kalabakan. (TIF)

S2 Fig Distribution of orangutan nests along 10km grid aerial transect in Deramakot. (TIF)

S3 Fig Distribution of orangutan nests along 10km grid aerial transect in Segama. (TIF)

S4 Fig Distribution of orangutan nests along 10km grid aerial transect in Kulamba. (TIF)

S5 Fig Distribution of orangutan nests along 10km grid aerial transect in Tabin. (TIF)

S6 Fig Distribution of orangutan nests along 10km grid aerial transect in Silabukan. (TIF)

S7 Fig Distribution of orangutan nests along 10km grid aerial transect in Trus Madi. (TIF)

S8 Fig Distribution of orangutan nests along 10km grid aerial transect in Trusan Sugut. (TIF)

S1 Table Population estimates in Sabah’s major orangutan population. (DOCX)

## References

[1] Ancrenaz M, Gumal M, Marshall AJ, Meijaard E, Wich SA, Husson, S. Pongo pygmaeus (errata version published in 2018). The IUCN Red List of Threatened Species 2016:e.T17975A123809220. 10.2305/IUCN.UK.2016-1.RLTS.T17975A17966347.en. (Downloaded on 25 January 2019). 2016.

[2] Utami-Atmoko S, Traylor-Holzer K, Rifqi MA, Siregar PG, Achmad B, Priadjati A, et al. (eds.) 2017. Orangutan population and habitat viability assessment: Final report. IUCN/SSC Conservation Breeding Specialist Group, Apple Valley, MN.

[3] Santika T, Ancrenaz M, Wilson KA, Spehar S, Abram N, Banes GL, et al First integrative trend analysis for a great ape species in Borneo. Scientific Reports. 2017; 7 (1): 4839 10.1038/s41598-017-04435-9 28687788 PMC5501861

[4] Voigt M, Wich SA, Ancrenaz M, Meijaard E, Abram N, Banes GL, et al Global demand for natural resources eliminated more than 100,000 Bornean orangutans. Current Biology. 2018; 28: 1–9. 29456144 10.1016/j.cub.2018.01.053

[5] Abram NK, Meijaard E, Wells JA, Ancrenaz M, Pellier AS, Runting RK, et al Mapping perceptions of species threats and population trends to inform conservation efforts: the Bornean orangutan case study. Diversity and Distributions. 2015; 21:487–499.

[6] Goosens B, Chikhi L, Ancrenaz M, Lackman-Ancrenaz I, Andau P, Bruford MW. Genetic signature of anthropogenic population collapse in orangutans. PLoS Biology. 2006; 4 (2): 0285–0291. 10.1371/journal.pbio.0040025 PMC1334199 16417405

[7] Meijaard E, Buchori D, Hadiprakarsa Y, Utami-Atmoko SS, Nurcahyo A, Tjiu A, et al Quantifying killing of orangutans and human-orangutan conflict in Kalimantan, Indonesia. PLoS ONE. 2011; 6 (11), 1–10. 10.1371/journal.pone.0027491 PMC3214049 22096582

[8] Wich SA, Gaveau D, Abram N, Ancrenaz M, Baccini A, Brend S, et al Understanding the impacts of land-use policies on a threatened species: Is there a future for the Bornean Orangutan? PLoS ONE. 2012; 7(11), 1–10. 10.1371/journal.pone.0049142 PMC3492325 23145100

[9] Gaveau DLA, Sloan S, Molidena E, Yaen H, Sheil D, Abram N, et al Four decades of forest persistence, clearance and logging in Borneo. PLoS ONE. 2014; 9(7). 10.1371/journal.pone.0101654 PMC4100734 25029192

[10] Gregory SD, Brook BW, Goosens B, Ancrenaz M, Alfred R, Ambu L, et al Long-term field data and climate-habitat models show that orangutan persistence depends on effective forest management and greenhouse mitigation. PloS ONE. 2012; 7(9). 10.1371/journal.pone.0043846 PMC3436794 22970145

[11] Wich S, Struebig M, Refisch J, Wilting A, Kramer-Schadt S, Meijaaard E. The Future of the Bornean Orangutan: impacts of change in land cover and climate. UNEP/ GRASP 2015.

[12] Struebig MJ, Fischer M, Gaveau D, Meijaard E, Wich SA, Gonner C, et al Anticipated climate and land-cover changes reveal refuge areas for Borneo’s orangutans. Global Change Biology. 2015; 21, 2891–2904. 10.1111/gcb.12814 25559092

[13] Davis JT, Mengersen K, Abram N, Ancrenaz M, Wells J, Meijaard E. It’s not just conflict that motivates killing of orangutans. PLoS ONE. 2013; 8(10). 10.1371/journal.pone.0075373 PMC3793980 24130707

[14] Mackinnon J. The behavior and ecology of wild orangutans (*Pongo pygmaeus*). Animal Behaviour. 1974; 22(1), 3–74.

[15] Davies G,Payne J. A faunal survey in Sabah. IUCN/WWF project No. 1692. WWF-Malaysia. 1982.

[16] Davies G. The orangutan in Sabah. Oryx. 1986; 20(1), 40–45.

[17] Payne J. Orangutan conservation in Sabah. Kuala Lumpur. WWF-Malaysia International. 1988; Report 3759. 137p.

[18] Ancrenaz M, Gimenez O, Ambu L, Ancrenaz K, Andau P, Goosens B, et al Aerial surveys give new estimates for orangutan in Sabah, Malaysia. PLoS Biology, 2005; 3(1): e3 10.1371/journal.pbio.0030003 15630475 PMC534813

[19] Reynolds G, Payne J, Sinun W, Mosigil G, Walsh RPD. Changes in forest landuse and management in Sabah, Malaysian Borneo, 1990–2010, with a focus on the Danum Valley region. Philosophical Transactions of the Royal Society. 2011; 366, 3168–3176. 10.1098/rstb.2011.0154 PMC3179641 22006960

[20] Sabah Forestry Department. Fact Sheets of Forest Reserves in Sabah. 2017; 5th ed. ISBN 978-983-42418-1-0. Malaysia. 48 pages.

[21] Ancrenaz M, Ambu L, Sunjoto I, Ahmad E, Manokaran K, Meijaard E, et al Recent surveys in the forests of Ulu-Segama Malua, Sabah, Malaysia, show that orangutans (*P*.*p*. *morio*) can be maintained in slightly logged forest. PLoS One. 2010; 5(7).24. 10.1371/journal.pone.0011510 PMC2901384 20634974

[22] Johns AD. Vertebrate response to selective logging: Implications for the design of logging systems. Philosophy Transactions of the Royal Society. Lond V Biol Sci. 1992; 335: 437–442.

[23] Husson SJ, Wich SA, Marshall AJ, Dennis RD, Ancrenaz M, Brassey, et al Orangutan distribution, density, abundance and impacts of disturbance In Wich SA, Utami-Atmoko S, Mitra Setia T, Van Schaik CP editors. Orangutans: geographic variation in behavioral ecology and conservation. OUP Oxford, 2010pp. 77–96.

[24] Ancrenaz M, Goosens B, Gimenez O, Sawang A, Lackman-Ancrenaz I. Determination of ape distribution and population size using ground and aerial surveys: a case study with orangutans in Lower Kinabatangan, Sabah, Malaysia. Animal Conservation. 2004; 7, 375–385.

[25] Kuehl H, Maisel F, Ancrenaz M, Williamson EA (eds). Best practice guidelines for surveys and monitoring of great ape populations. Gland, Switzerland: IUCN/SSC Primate Specialist Group. 2008; 32 pp.

[26] R Core Team. R: A language and environment for statistical computing. R Foundation for statistical Computing, Vienna, Austria. 2014; URL http://www.R-project.org/.

[27] Burke C, Rashman MF, Longmore SN, McAree O, Glover-Kapfer P, Ancrenaz M, et al. Successful observation of orangutans in the wild with thermal-equipped drones. Journal of Unmanned Vehicle Systems. 10.1139/juvs-2018-0035.

[28] Abram NK, Ancrenaz M. Orangutan, oil palm and RSPO: Recognizing the importance of the threatened forests of the Lower Kinabatangan, Sabah, Malaysian Borneo. Ridge to Reef, Living Landscape Alliance, Borneo Futures, Hutan, and Land Empowerment Animals People. Kota Kinabalu, Sabah, Malaysia. 2017.

[29] Husson SJ, Morrogh-Bernard H, Santiano, Purwanto A, Harsanto FA, McLardy C, et al. Bornean orangutans in the Sabangau peat-swamp forest. In: Lanjouw A, Rainer H, White A. (editors) State of the Apes: Industrial agriculture and ape conservation. The Arcus Foundation, New York. 2015. 200–207.29.

[30] Brodie J, Paxton M, Nagulendran K, Balamurugan G, Clements GR, Reynolds G, et al Science, policy and implementation for landscape-scale habitat connectivity. Society for Conservation Biology. 2016; 30(5): 950–961. 10.1111/cobi.12667 26648510

