# Supplementary figures and images for: Changes to Sabah’s orangutan population in recent times: 2002–2017
Source: PLoS One. 2019 Jul 17;14(7):e0218819. doi: 10.1371/journal.pone.0218819 (PMC6636716; doi:10.1371/journal.pone.0218819)

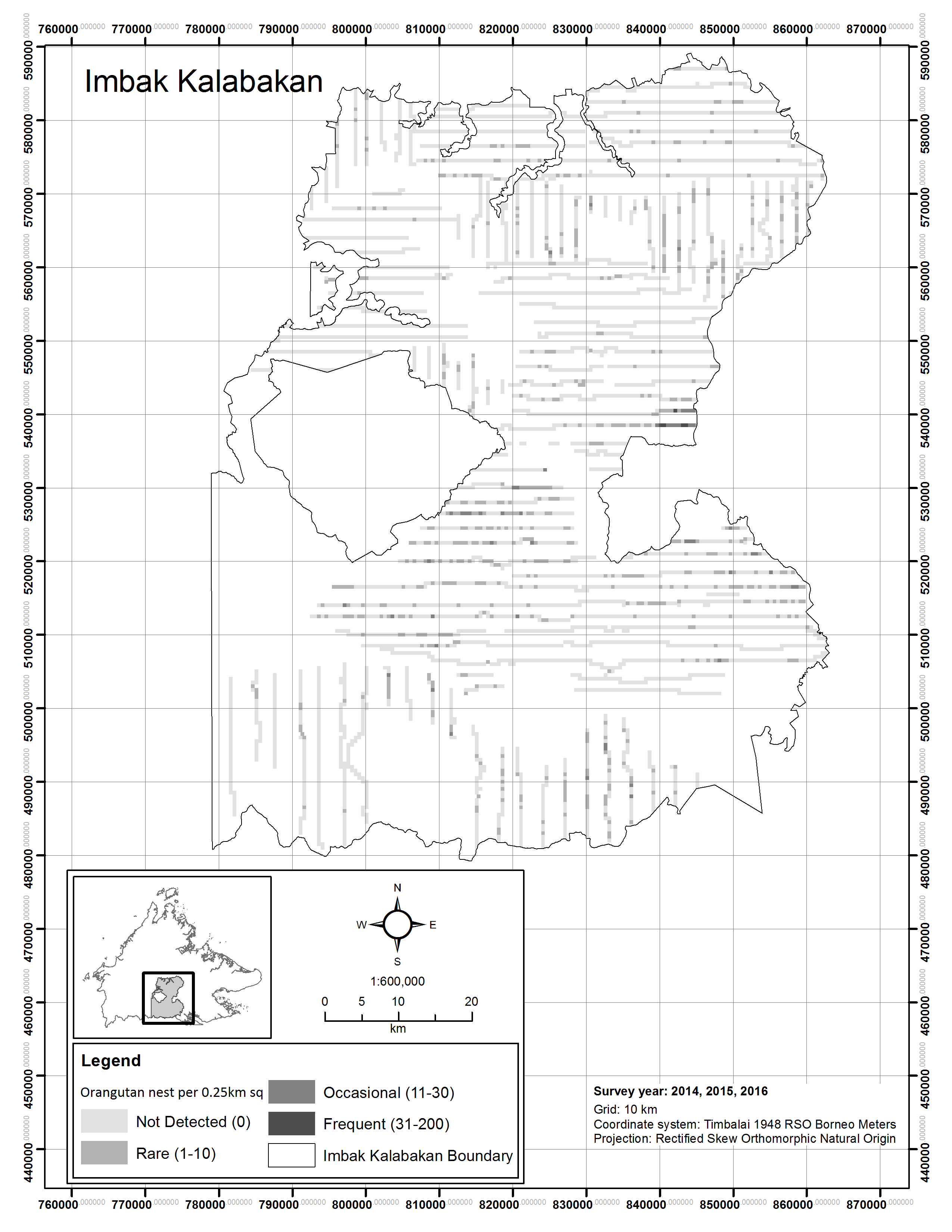

Supplement: S1 Fig — (TIF) [file pone.0218819.s001.tif]

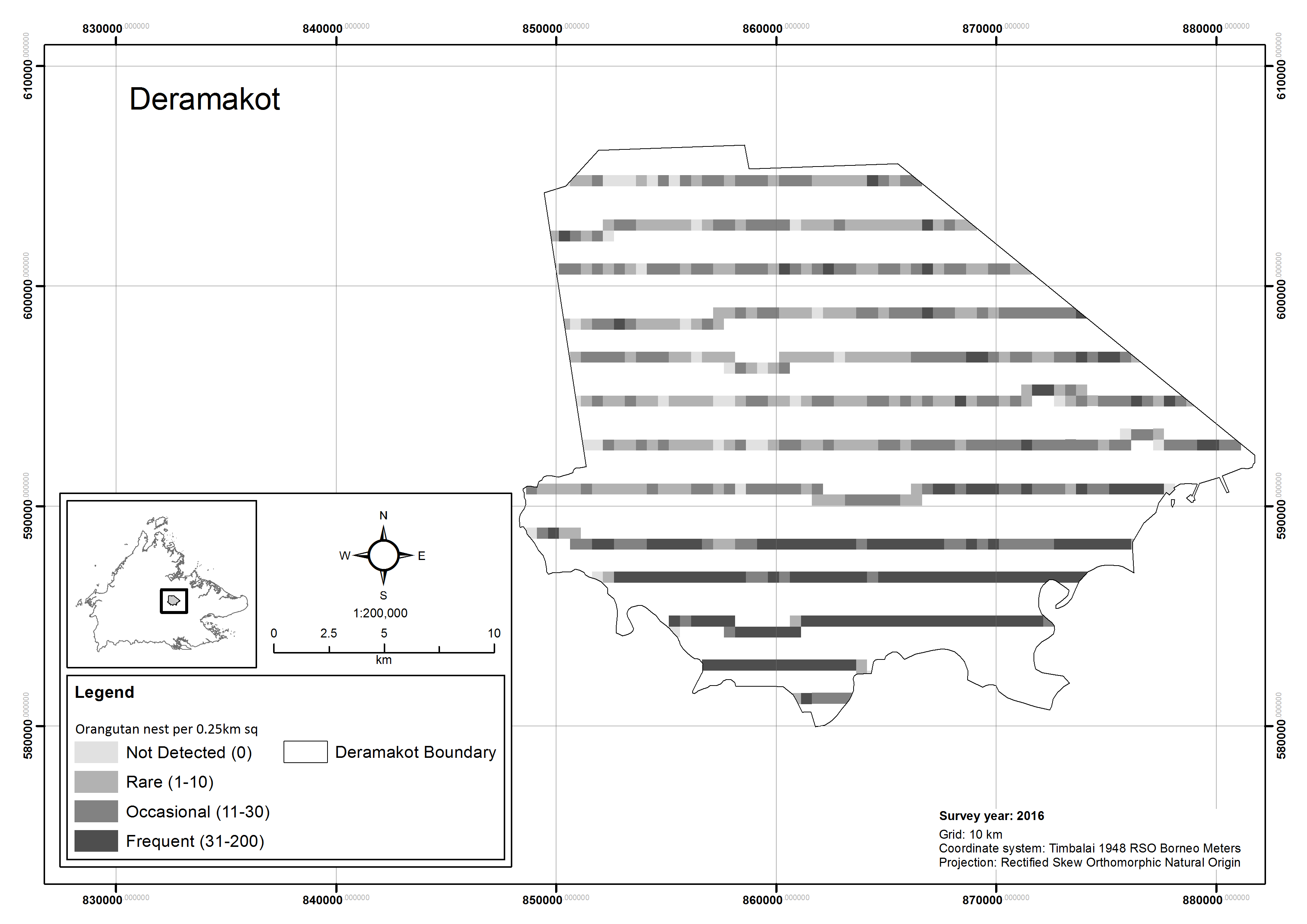

Supplement: S2 Fig — (TIF) [file pone.0218819.s002.tif]

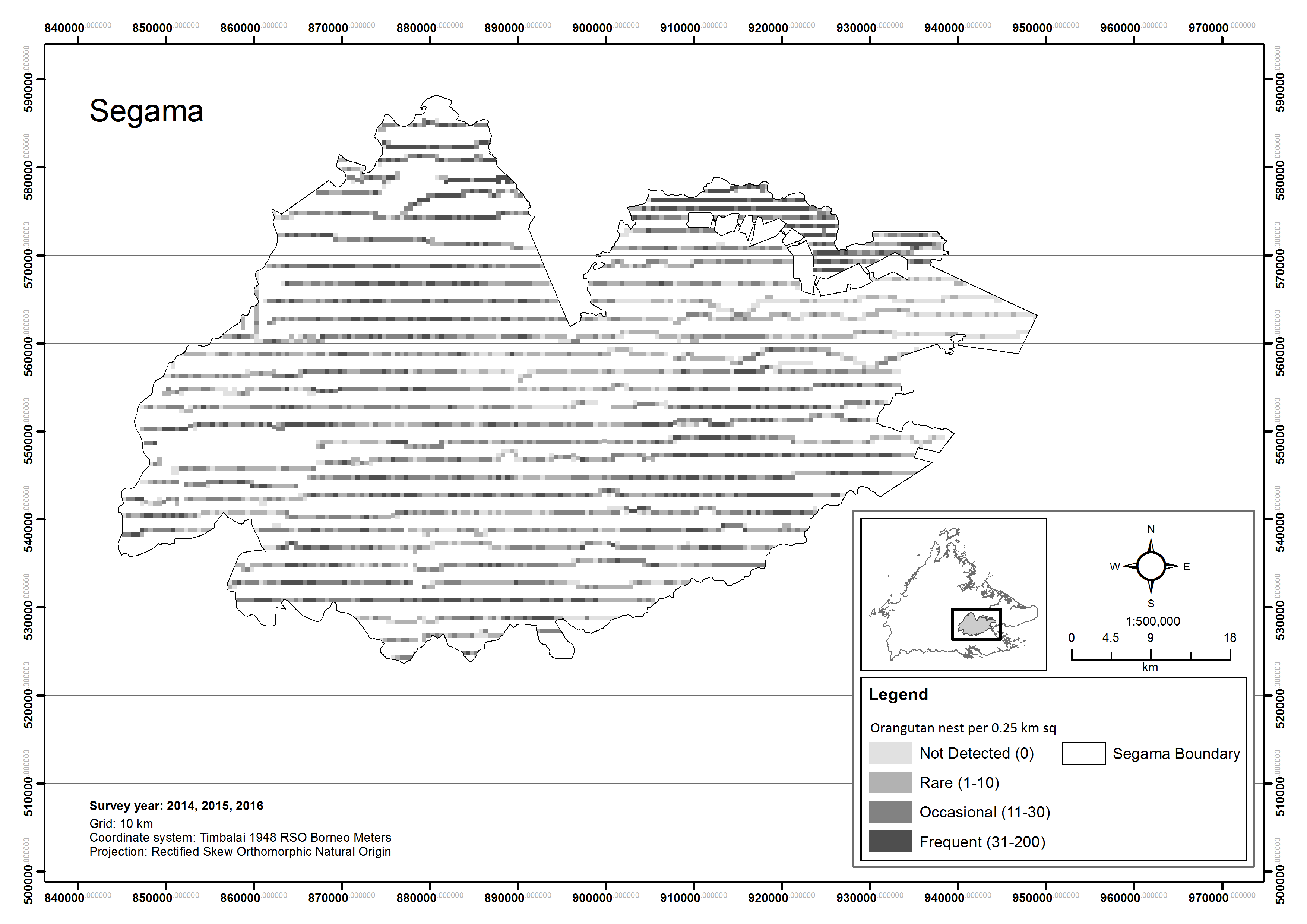

Supplement: S3 Fig — (TIF) [file pone.0218819.s003.tif]

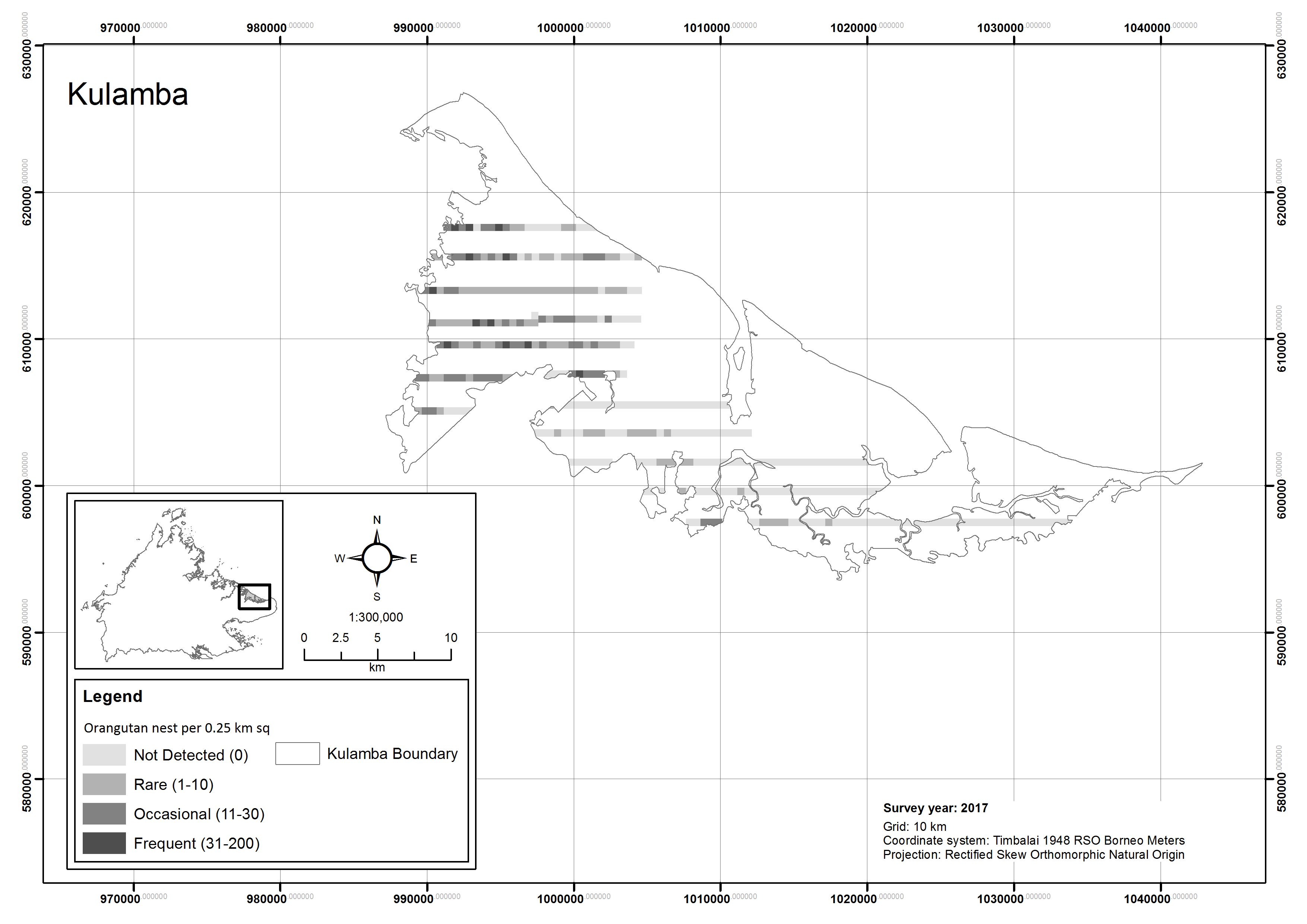

Supplement: S4 Fig — (TIF) [file pone.0218819.s004.tif]

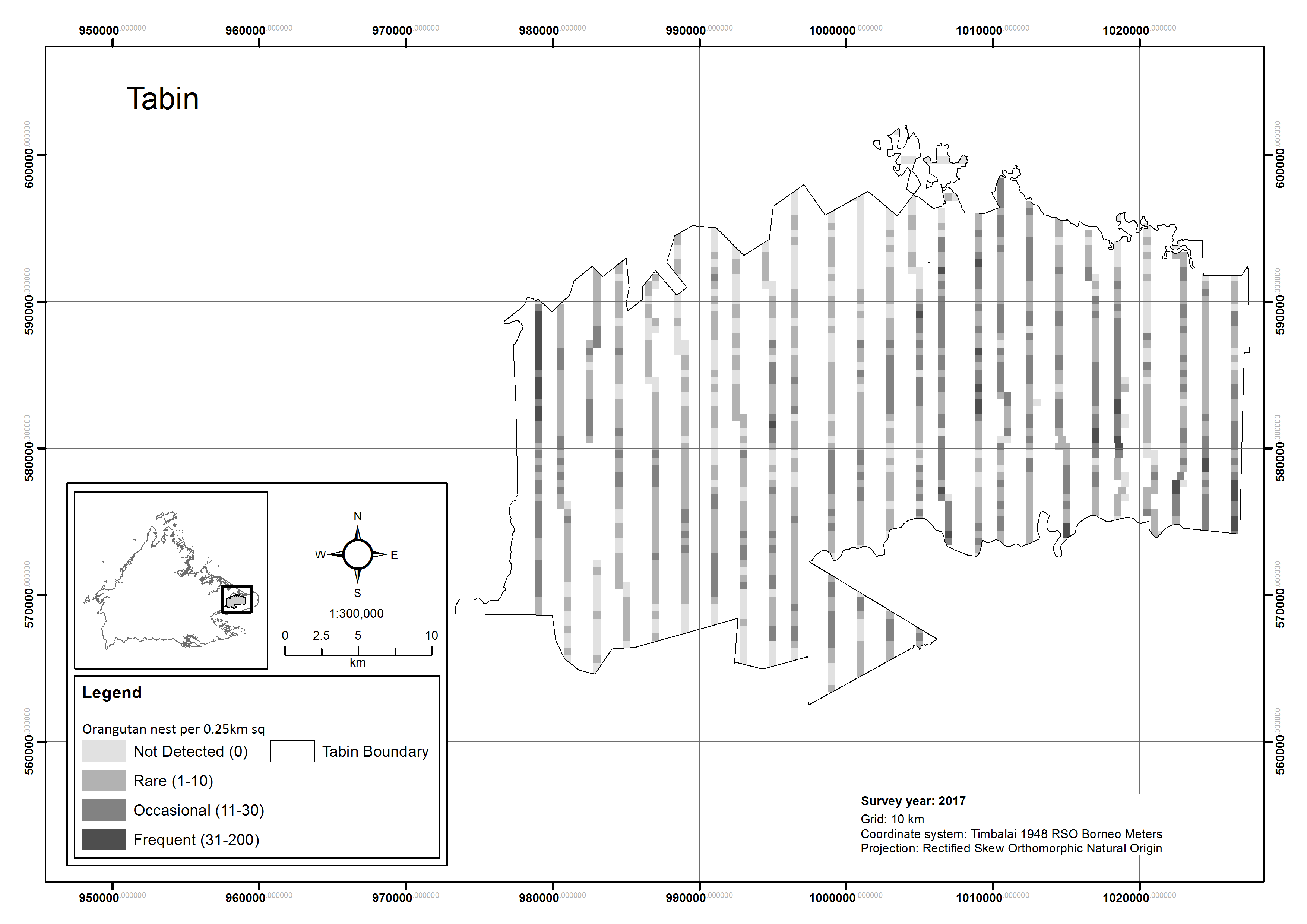

Supplement: S5 Fig — (TIF) [file pone.0218819.s005.tif]

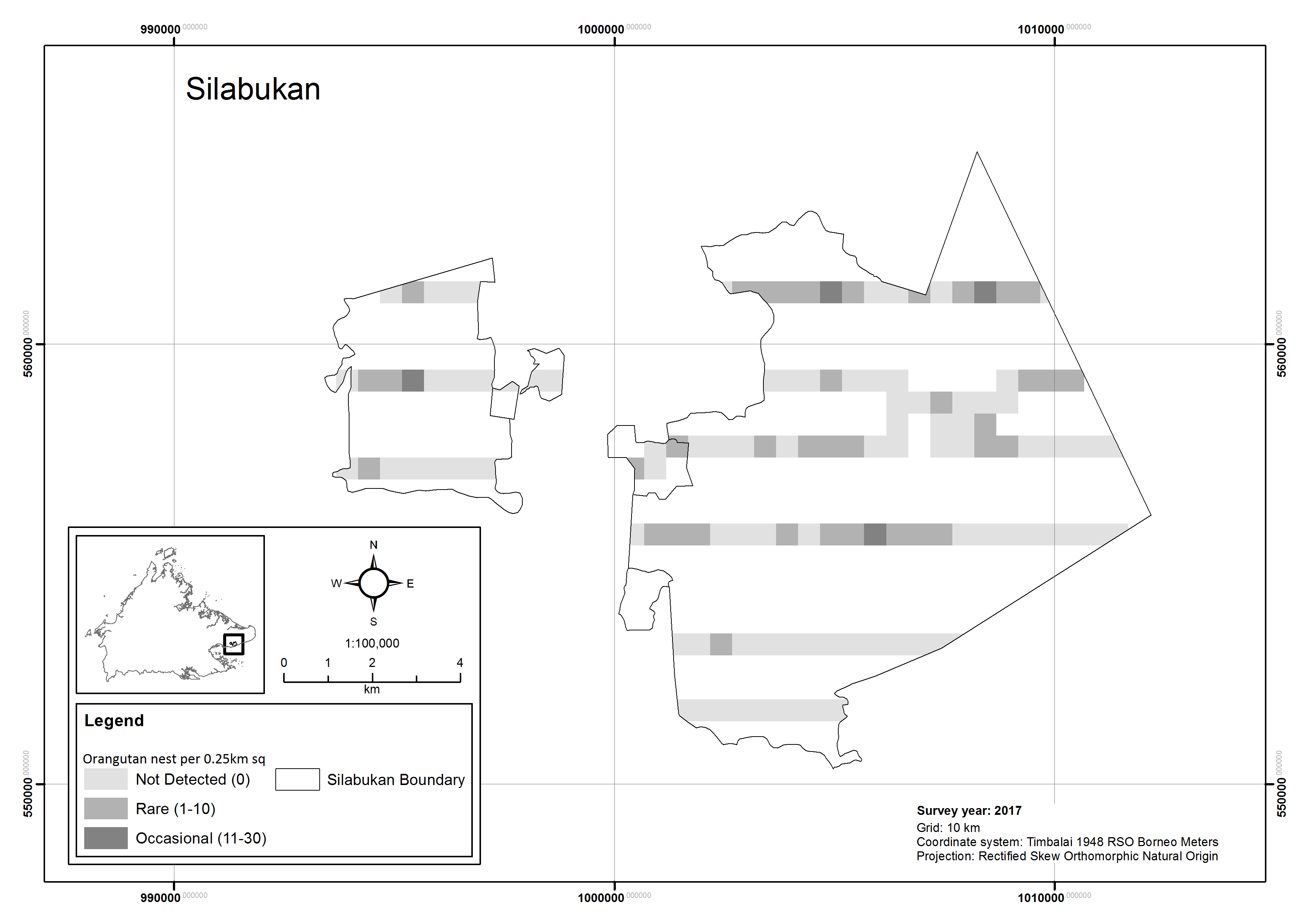

Supplement: S6 Fig — (TIF) [file pone.0218819.s006.tif]

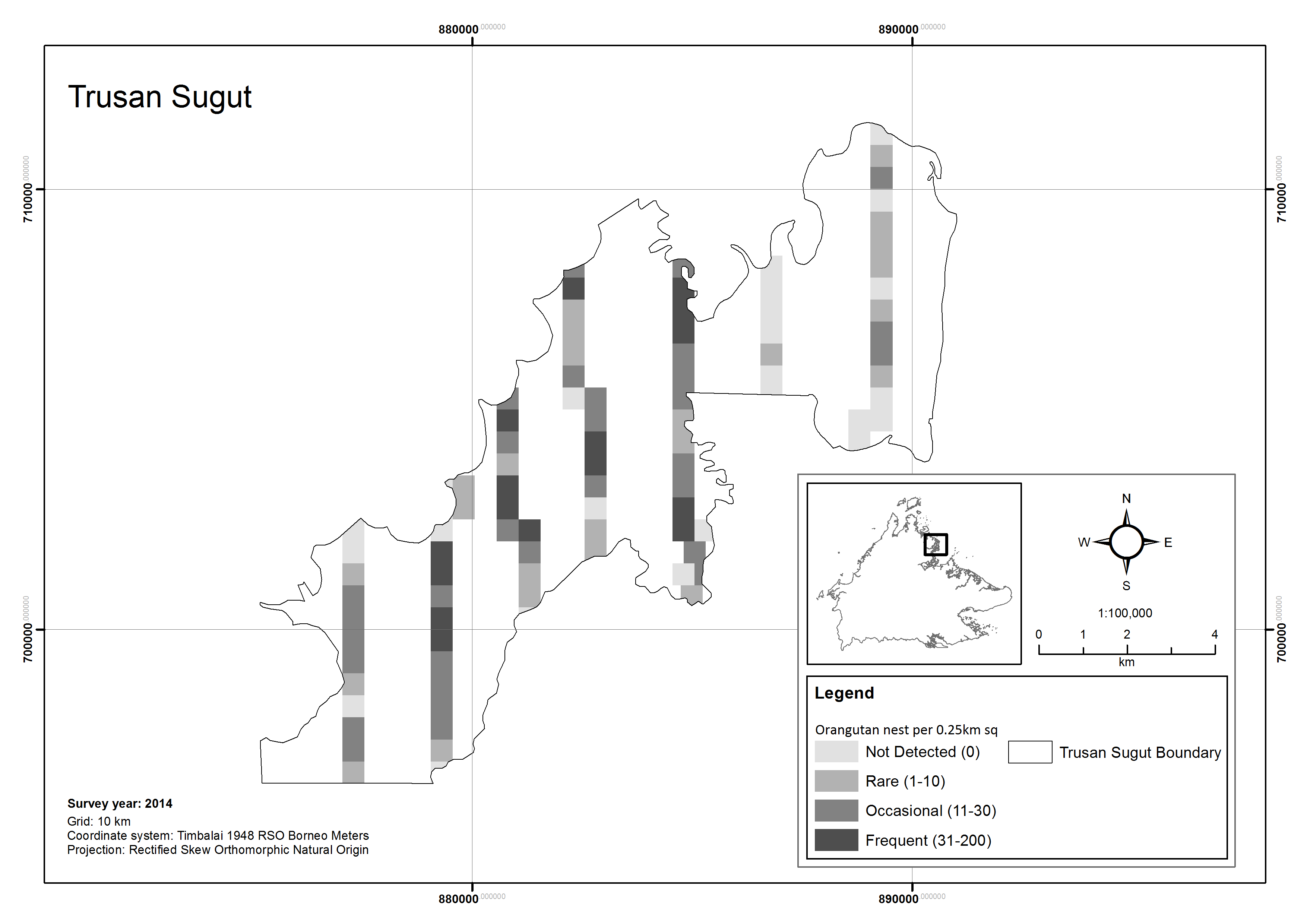

Supplement: S7 Fig — (TIF) [file pone.0218819.s007.tif]

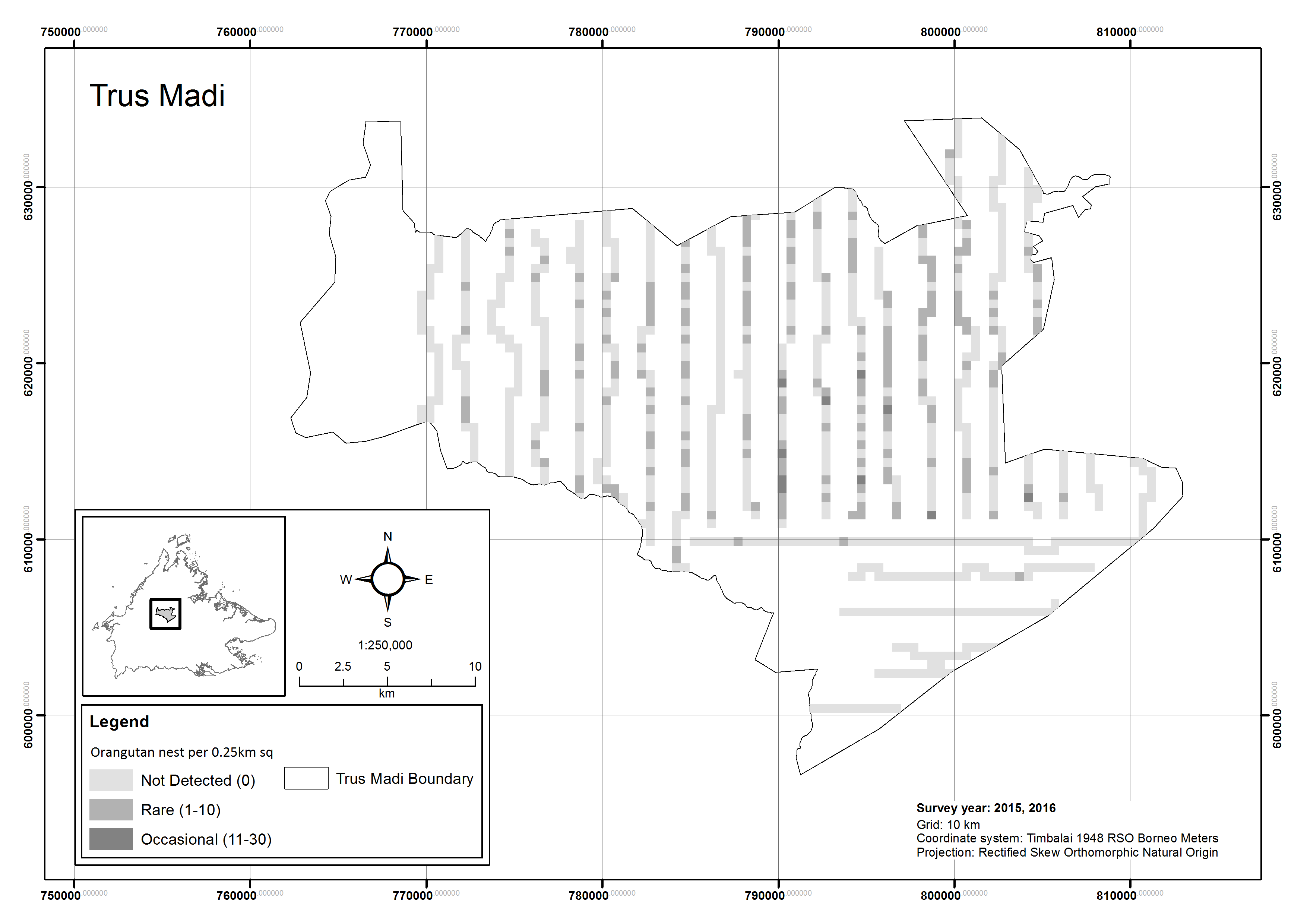

Supplement: S8 Fig — (TIF) [file pone.0218819.s008.tif]
