# Supplementary material for: Changes to Sabah’s orangutan population in recent times: 2002–2017
Source: PLoS One. 2019 Jul 17;14(7):e0218819. doi: 10.1371/journal.pone.0218819 (PMC6636716; doi:10.1371/journal.pone.0218819)
Supplement: S1 Table — (DOCX) [file pone.0218819.s009.docx]

| **Forest Reserve** | **Survey year** | **Area (km^2^)** | **OU per km^2^** | **Population size** |
| --- | --- | --- | --- | --- |
| **National parks:** |  |  |  |  |
| Kinabalu | Mid 80s [17] | 200 | n.a | 50 (27-75) |
| Crocker Range | 2002-03 [18] | 900 | 0.2 (0.07-0.59) | 181 (62-258) |
| **Wildlife Reserve/ Sanctuary:** |  |  |  |  |
| Kulamba | 2014-17 | 203.83 | 1.20 (0.81-1.78) | 245(165-364) |
| Tabin | 2014-17 | 1123.96 | 1.06 (0.72-1.58) | 1195(805-1775) |
| Lower Kinabatangan | 2017 [29] | 410 | na | 800 |
| **Class I-Protection forest/ Class VII-Virgin Jungle Reserve** | | |  |  |
| Trusan Sugut | 2014-17 | 85.34 | 1.38 (0.93-2.05) | 118 (79-175) |
| Nuluhon Trus Madi | 2014-17 | 41.93 | 0.20 (0.13-0.30) | 8 (5-13) |
| Bukit Taviu | 2014-17 | 86.17 | 0.46 (0.31-0.69) | 40 (26-60) |
| Northern Kuamut (West) | 2014-17 | 504.69 | 0.38 (0.25-0.58) | 193 (128-292) |
| Sg Imbak Bufferzone | 2014-17 | 192.26 | 0.32 (0.21-0.49) | 62 (41-94) |
| Sg Lulunguyon | 2014-17 | 20.58 | 0.45 (0.30-0.68) | 9 (6-14) |
| Sg Ayop | 2014-17 | 15.5 | 0 | 0 |
| Imbak Canyon | 2014-17 | 133.61 | 0.18 (0.11-0.27) | 24 (15-37) |
| Mt Magdalena (West) | 2014-17 | 350.91 | 0.55 (0.37-0.82) | 192 (128-287) |
| Gunung Rara Wildlife Corridor | 2014-17 | 89.22 | 0.16 (0.10-0.25) | 14 (9-22) |
| Sg Tiagau and Ext | 2014-17 | 269.16 | 0.36 (0.24-0.54) | 97 (64-146) |
| Tambulanan | 2014-17 | 32.77 | 0.14 (0.09-0.21) | 4 (3-7) |
| Sg Anjeran Jemut | 2014-17 | 37.5 | 0.13 (0.08-0.20) | 5 (3-7) |
| Sg Sumagas | 2014-17 | 41.96 | 0.22 (0.15-0.34) | 9 (6-14) |
| Maliau Buffer | 2014-17 | 344.62 | 0.40 (0.27-0.60) | 138 (91-207) |
| Northern Gunung Rara | 2014-17 | 58.85 | 1.37 (0.92-2.03) | 81 (54-120) |
| Malua | 2014-17 | 339.54 | 1.76 (1.18-2.61) | 597 (402-888) |
| Ulu Segama | 2014-17 | 1272.35 | 1.28 (0.87-1.91) | 1634 (1101-2426) |
| Danum Valley | 2014-17 | 438.68 | 1.37 (0.92-2.03) | 601 (405-892) |
| Mt Magdalena (East) | 2014-17 | 132 | 1.28 (0.87-1.91) | 170 (114-252) |
| Mt Louisa | 2014-17 | 642.36 | 1.37 (0.92-2.03) | 880 (593-1307) |
| Bukit Piton | 2014-17 | 121.64 | 1.44 (0.97-2.14) | 176 (119-261) |
| Sg Taliwas | 2014-17 | 97.13 | 1.20 (0.81-1.78) | 116 (78-173) |
| Northern Kuamut (East) | 2014-17 | 385.55 | 1.08 (0.73-1.60) | 417 (281-619) |
| Kuala Meruap | 2014-17 | 183.71 | 0.45 (0.30-0.68) | 83 (55-124) |
| Mt Hatton | 2014-17 | 89.68 | 0.92 (0.62-1.37) | 82 (55-123) |
| Silabukan | 2014-17 | 104.95 | 0.48 (0.32-0.72) | 51 (34-76) |
| Nurod-Urod | 2014-17 | 16.51 | 0.23 (0.15-0.35) | 4 (2-6) |
| Sg Imbak | 2014-17 | 126.18 | 0.32 (0.21-0.48) | 40 (26-61) |
| Ulu Kalumpang | 2002-03 [18] | 480 | 0.30 (0.11-0.85) | 144 (54-408) |
| Tawai | 2002-03 [18] | 210 | 0.07 (0.02-0.23) | 15 (5-49) |
| Sepilok | 2002-03 [18] | 40 | n.a | 200 (100-300) |
| Tangkulap | 2002-03 [18] | 350 | 0.62 (0.23-1.70) | 217 (79-594) |
| **Class II- Commercial forest** |  |  |  |  |
| Trus Madi | 2014-17 | 676.09 | 0.40 (0.27-0.61) | 272 (181-410) |
| Deramakot | 2014-17 | 550.83 | 1.61 (1.08-2.39) | 887 (597-1318) |
| Kuamut (West) | 2014-17 | 72.86 | 0.28 (0.18-0.43) | 20 (13-31) |
| Sg Pinangah | 2014-17 | 390.87 | 0.21 (0.14-0.33) | 84 (55-129) |
| Gunung Rara | 2014-17 | 601.48 | 0.40 (0.27-0.60) | 240 (160-362) |
| Kalabakan | 2014-17 | 981.99 | 0.43 (0.29-0.65) | 424 (282-637) |
| Sapulut | 2014-17 | 583.07 | 0.18 (0.12-0.28) | 107 (69-165) |
| Kuamut (East) | 2014-17 | 203.56 | 1.33 (0.90-1.97) | 271 (182-402) |
| Lingkabau | Mid 80s [17] | 300 | n.a | 100 (75-150) |
| Ulu Tungud | 2002-03 [18] | 720 | 0.04 (0.01-0.14) | 29 (9-99) |
| Segaliud-Lokan | 2002-03 [18] | 580 | 1.19 (0.44-3.23) | 692 (255-1874) |
